# *Protomedetera*, a new genus from the Oriental and Australasian realms (Diptera, Dolichopodidae, Medeterinae)

**DOI:** 10.3897/zookeys.743.22696

**Published:** 2018-03-14

**Authors:** Chufei Tang, Patrick Grootaert, Ding Yang

**Affiliations:** 1 Department of Entomology, College of Plant Protection, China Agricultural University, Beijing 100094, China; 2 Entomology, Royal Belgian Institute of Natural Sciences, Vautierstraat 29, B-1000 Brussels, Belgium; 3 National Biodiversity Centre, NParks and Lee Kong Chian Natural History Museum, National University of Singapore, Singapore

**Keywords:** Australasia, Medeterinae, new genus, Oriental

## Abstract

*Protomedetera* **gen. n.** (Diptera: Dolichopodidae), a new genus of the subfamily Medeterinae, is described from the Oriental and Australasian realms based on four new species. *Protomedetera singaporensis* Grootaert & Tang, **sp. n.** is designated as type of the new genus. The genus is peculiar because of the small body size, the small globular first flagellomere (postpedicel), the simple male genitalia with indistinct or small epandrial lobe and half-hidden cercus. The following four new species are described and illustrated: *P. biconvexa* **sp. n.**, *P. biseta* **sp. n.**, *P. glabra* **sp. n.**, and *P. singaporensis* **sp. n.** A key to the species of the new genus is provided.

## Introduction

Medeterinae is a wide spread dolichopodid subfamily which occurs in all geographical realms. Overall, there have been 25 extant genera reported, with three new genera *Nikitella*, *Medeterella*, and *Demetera* defined by Grichanov (2011). The reported species diversity and generic diversity in the Oriental and Australasian regions have relatively lower records than the other geographic regions (Yang et al. 2006, Yang et al. 2011). In the present paper the new genus *Protomedetera* gen. n. is found in both the Oriental region and Australasian realms with four new species: *P. biconvexa* sp. n., *P. biseta* sp. n., *P. glabra* sp. n. and *P. singaporensis* sp. n. The genus is found in four different countries: Papua New Guinea, Singapore, Malaysia, and Indonesia. Hence, it is potentially present all over the Oriental and major parts of Australasia.

## Material and methods

The specimens on which this study is based were partly collected by Dr Olivier Missa in Papua New Guinea, by fogging tree canopies in a primary rain forest in Baiteta (4°59'10.36"S, 145°45'47.05"E; Madang province). This material was first preserved in salt that caused bleaching of the flies. Later they were transferred to 75% ethanol. The Oriental material (Singapore, Malaysia) was collected by hand on tree trunks or rarely in a Malaise trap. The type material is conserved in the collections of the Royal Belgian Institute of Natural Sciences (**RBINS**) in Brussels or in the Lee Kong Chian Natural History Museum (**LKCNHM**), Singapore as indicated.

Morphological terminology for adult structures mainly follows McAlpine (1981), and the structures of the male genitalia follow Cumming and Wood (2009). Photographs were taken with a Canon EOS 600D camera at the Royal Belgian Institute of Natural Sciences and then stacked by Helicon Focus 6.0.

The acquisition of the NGS COI barcode of *P. singaporensis* sp. n. follows the PCR conditions and the primer from Meier et al. (2016).

The following abbreviations are used:

**acr** acrostichal bristle (s),

**ad** anterodorsal bristle (s),

**av** anteroventral bristle (s),

**dc** dorsocentral bristle (s),

**npl** notopleural bristle (s),

**pd** posterodorsal bristle (s),

**oc** ocellar bristle (s),

**ph** posthumeral bristle,

**pp** propleuron,

**pt** postalar bristle (s),

**pv** posteroventral bristle (s),

**sa** supraalar bristle (s),

**sc** scutellar bristle (s),

**su** sutural bristle (s),

**vt** verticle bristle(s),

**CuAx ratio** length of dm-cu / length of distal portion of CuA,

**LI** fore leg,

**LII** mid leg,

**LIII** hind leg.

## Taxonomy

### Protomedetera gen. n.

Taxon classificationAnimaliaDipteraDolichopodidae

http://zoobank.org/D8C426F6-F48A-47C0-942B-E8424868BFE6

#### Type species.

*Protomedetera singaporensis* Grootaert & Tang, sp. n.

#### Etymology.

The name *Protomedetera* gen. n. is proposed since this genus looks like a small and simplified *Medetera*.

#### Diagnosis.

Body small and stout, body length less than or equal to 2.0 mm. Eye without soft hair between each facet. Two oc, two vt. Scape short and small, almost invisible in lateral view. Pedicel cup-like, large, with first flagellomere sunken in it; usually with long apical bristles over first flagellomere. First flagellomere rounded, wider than long or nearly as long as wide, with short pubescence. Arista nearly bare or shortly pubescent, basal portion of arista very short. Ocellar tubercle distinct but not strongly raised. Upper postcranium deeply concave. Proboscis not strongly sclerotized.

Thorax raised dorsally at shoulder. Mesonotum flat before scutellum. Two h, one ph, one su, two npl, two sa, one pt, five to six dc, biseriate acr. Anterior portion sometimes densely covered by short bristles. Legs only with few bristles, wholly yellow or mainly yellow including tarsomeres. Hind coxa with one weak outer bristle. Hind tibia without any ad/pd/av/pv. Hind tarsomere I short and flat. Wing nearly hyaline, tinged light yellow; veins light brown, R_4+5_ and M parallel. CuAx ratio no more than 0.25. Squama pale with long pale hairs. Halter pale.

Hypopygium: Epandrium distinctly longer than wide, nearly triangular. Epandrial lobe indistinct or small. Surstylus simple. Cercus half hidden in epandrium, with marginal bristles, covered by short peg-like bristles. Phallus usually hidden in hypandrium, sometimes modified apically.

#### Key to males of *Protomedetera* gen. n.

**Table:** 

1	Pedicel with long apical bristles, longer than total length of pedicel and flagellomere	**2**
–	Pedicel without long apical bristles	***P. glabra* sp. n.**
2	Basal third of costa thickened; cercus (Fig. 2) short, thick, with two round apical papilla-like structures each with a strong apical bristle	***P. biconvexa* sp. n.**
–	Basal third of costa not thickened; cercus simple, without apical papillae	**3**
3	Width of face at middle 1.4 times as wide as first flagellomere. Pedicel with only one long apical bristle distinctly longer than total length of pedicel and first flagellomere (Fig. 9). Cercus (Fig. 10c) nearly oval, 3.2 times as long as wide, slightly narrow at tip, with row of long marginal bristles at apical half, covered by short weak peg-like bristles	***P. singaporensis* sp. n.**
–	Width of face at middle as wide as first flagellomere. Pedicel with two long apical bristles 1.5 times as long as the total length of pedicel and first flagellomere (Fig. 3). Fore coxa yellow. Cercus (Fig. 4c) nearly oval, 3.6 times as long as wide, narrow at tip, with several long apical marginal bristles and some short peg-like bristles	***P. biseta* sp. n.**

### Protomedetera bico nvexa sp. n.

Taxon classificationAnimaliaDipteraDolichopodidae

http://zoobank.org/96A323FB-A8C7-40E4-A22F-D65864E107B5

Figs 1 , 2

#### Diagnosis.

Pedicel with four apical bristles twice as long as total length of first flagellomere and pedicel. Vein costa bold at basal 1/3. Cercus short thick, with two round apical protuberances each with a strong apical bristle. Hypandrium with one irregular process at apical 2/5 to 1/4, sharp apically. Phallus not modified.

#### Description.

**Figure 1.**
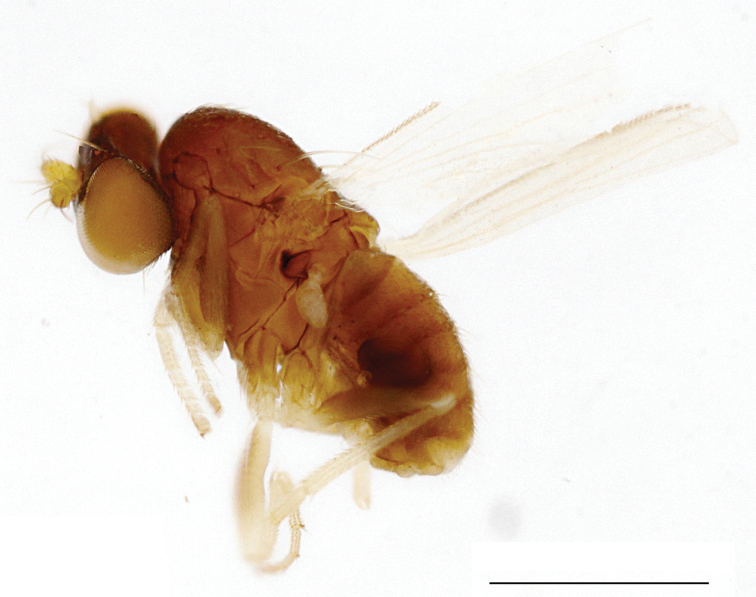
*Protomedetera biconvexa* sp. n. holotype male, habitus, lateral view. Scale bar: 1 mm (photo credit Ms Chufei Tang).

***Male*** (Fig. 1). Body length 1.7 mm, wing length 1.5 mm, wing width 0.7 mm.

*Head* metallic green, nearly black, with grey pollinosity; eyes separated; face nearly parallel, width of face at middle as wide as first flagellomere. Hairs and bristles on head black, postocular bristles and posteroventral hairs short pale. Two strong oc, two strong vt. Antenna wholly yellow; scape short and small, almost invisible; pedicel cup-like, large, with first flagellomere sunken in it, with circlet of apical bristles of which four very long strong, distinctly covering first flagellomere, twice as long as total length of first flagellomere and pedicel; first flagellomere small, rounded, nearly as long as wide, with tiny pubescence; arista black, almost bare, nearly as long as head width, with basal segment 0.1 times as long as apical segment. Ocellar tubercle distinct but not strongly raised. Upper postcranium deeply concave. Proboscis yellow, not strongly sclerotized, with light thin lines and short strong apical bristle; palpus yellow, slightly raised, rounded, with black preapical bristle.

*Thorax* raised dorsally at anterior area, dark metallic green, with some grey pollinosity. Mesonotum flat before scutellum. Pp with one strong spine-like bristle at lower 1/3. Hairs and bristles on thorax black; two h, one ph, one su, two npl, two sa, one pt, five dc, six biseriate acr at anterior portion. Anterior portion densely covered by short bristles. Scutellum with one pair of strong sc.

*Legs* mainly yellow except mid and hind coxae, dorsum of mid and hind femora brown. Hairs and bristles on legs mainly black. One weak prothoracic bristle above fore coxa. Fore coxa at a distance with mid coxa. Mid and hind coxae each with one weak outer bristle at middle. Femora without distinct bristles. Mid tibia with one ad at middle and three apical bristles. Hind tibia with two tiny apical bristles. Hind tarsomere I somewhat shortened and flat. Tibiae and five tarsomeres of legs LI : 2.0 : 1.0 : 0.3 : 0.3 : 0.2 : 0.4; LII : 3.0 : 1.5 : 0.8 : 0.5 : 0.3 : 0.4 ; LIII : 3.5 : 0.8 : 1.0 : 0.8 : 0.4 : 0.5. Wing nearly hyaline, tinged light yellow; veins light brown, costa vein thickened at basal 1/3, R_4+5_ and M parallel. CuAx ratio 0.25. Squama pale with long pale hairs. Halter pale.

**Figure 2.**
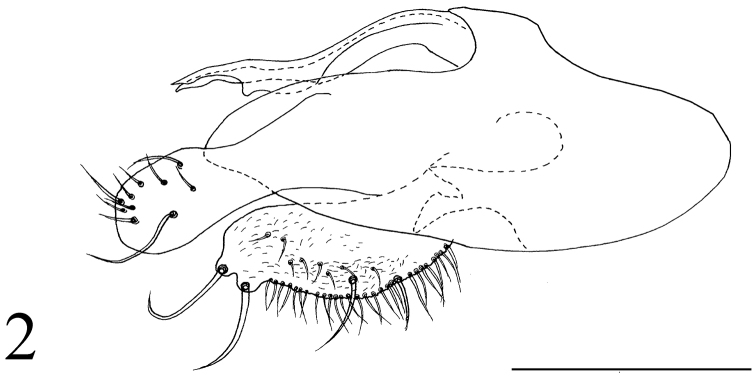
*Protomedetera biconvexa* sp. n. holotype male terminalia. Scale bar: 0.1 mm.

*Abdomen* metallic green with grey pollinosity. Hairs and bristles black. Hypopygium (Fig. 2): Epandrium yellowish. Hairs and bristles pale. Epandrium nearly rectangular, twice as long as wide; epandrial lobe indistinct. Surstylus wide, finger-like, rounded at tip, with ten bristles, of which one extremely long. Cercus short thick, with two round apical protuberances each with one long strong apical bristle. Hypandrium with one irregular process at apical 2/5 to 1/4, sharp at tip. Phallus normal.

***Female.*** Unknown.

#### Etymology.

The name *biconvexa* means two protuberances, referring to the two apical two protuberances of the cercus.

#### Material examined.

Holotype male, PAPUA NEW GUINEA, Madang province, Baiteta (4°59'10.36"S, 145°45'47.05"E) FOG XH, 16/III/1994, leg. Olivier Missa (coll. RBINS).

Paratypes: PAPUA NEW GUINEA, Madang province, Baiteta (4°59'10.36"S, 145°45'47.05"E), FOG AR 15, 15/VI/1995, leg. Olivier Missa; 1 male, FOG AR 24, 5/VII/1995; 1 male, FOG XJ, 7/IV/1994 (coll. RBINS). MALAYSIA: 1 male, Pulau Tioman, Salang, 13/VII/ 2005 (leg. I. Van de Velde) (coll. RBINS).

### Protomedetera biseta sp. n.

Taxon classificationAnimaliaDipteraDolichopodidae

http://zoobank.org/EEF27383-0296-46B0-A26B-52CD9F9520C6

Figs 3 , 4

#### Diagnosis.

Pedicel with two long apical bristles 1.5 times as long as total length of pedicel and first flagellomere. Cercus nearly oval, 3.6 times as long as wide, narrow at tip, with several long apical marginal bristles and some short peg-like bristles. Hypandrium simple, rounded apically in lateral view. Phallus thin, hook-like apically, with a preapical denticle.

#### Description.

**Figure 3.**
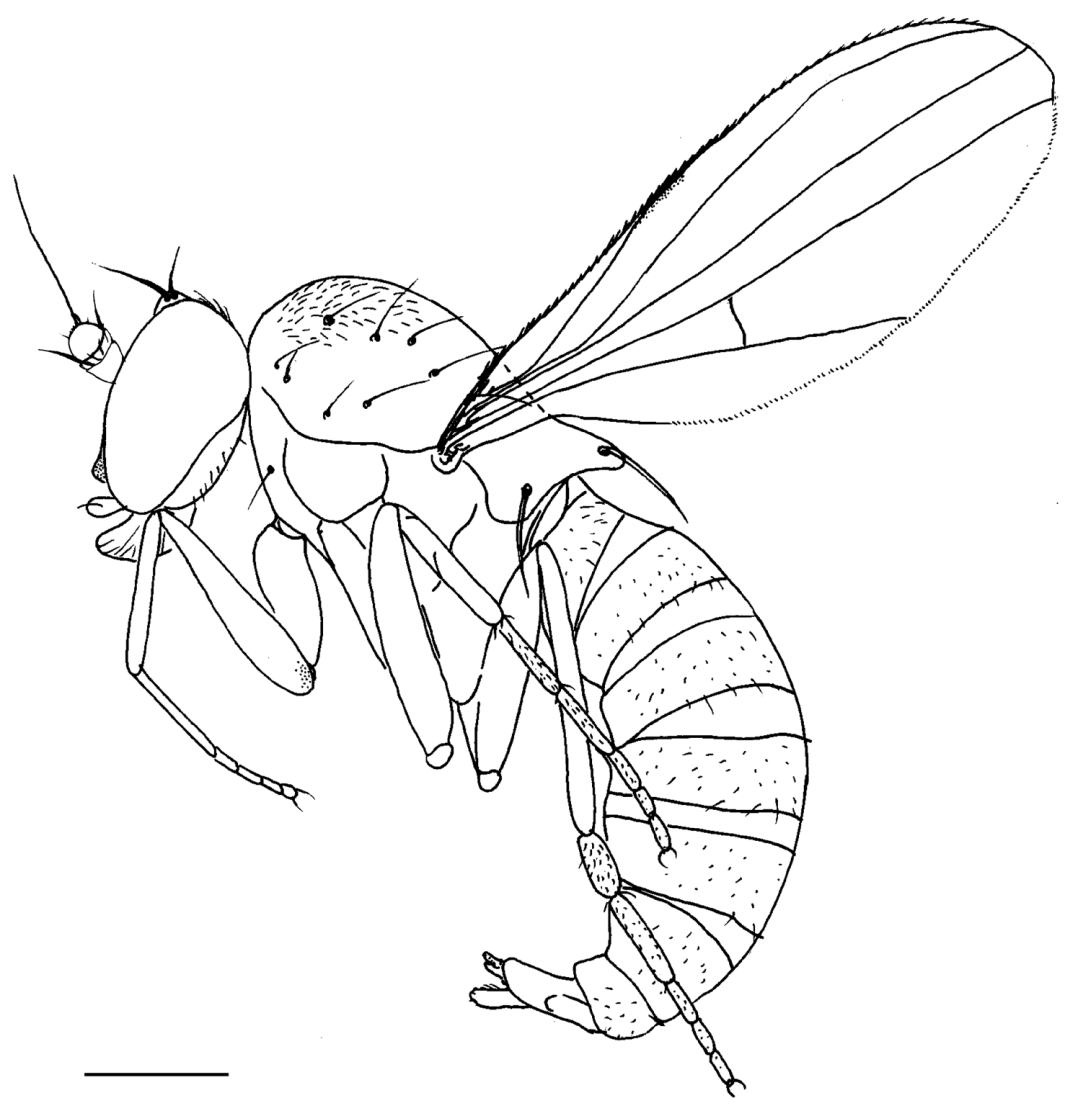
*Protomedetera biseta* sp. n. holotype male, habitus, lateral view Scale bar: 1 mm (photo credit Ms Chufei Tang).

***Male*** (Fig. 3). Body length 2.0 mm, wing length 1.7 mm, wing width 1.2 mm.

*Head* metallic green, nearly black, with grey pollinosity; eyes separated; face nearly parallel, width of face at middle as wide as first flagellomere. Hairs and bristles on head black, postocular bristles and posteroventral hairs short and pale. Two strong oc, two strong vt. Scape and pedicel yellow; scape short and small, almost invisible; pedicel cup-like, large, with first flagellomere sunken in it, with circlet of apical bristles of which two bristles 1.5 times as long as total length of pedicel and first flagellomere; first flagellomere small, rounded, brown, nearly as long as wide, with tiny pubescence; arista black, almost bare, nearly as long as head, with basal segment extremely short, less than 0.1 times as long as apical segment. Ocellar tubercle distinct but not strongly raised. Upper postcranium deeply concave. Proboscis yellow, not strongly sclerotized, with light thin lines; palpus yellow, slightly raised, rounded, with yellow preapical bristle.

*Thorax* raised dorsally at front area, dark metallic green, with some grey pollinosity. Mesonotum flat before scutellum. Hairs and bristles on thorax pale; two h, one ph, one su, two npl, two sa, one pt, six dc, five biseriate acr at anterior 1/2. Shoulder portion covered by short bristles. Scutellum with one pair of strong sc.

*Legs* mainly yellow except mid and hind coxae brown. Hairs and bristles on legs mainly pale. One weak prothoracic bristle above fore coxa. Fore coxa with row of bristles anteriorly. Hind coxa with one weak outer bristle. Fore femur with six pv (three at basal 1/6, three at apical 1/6). Hind femur with four pv at apical 1/4. Mid tibia with one ad at basal 1/3 and three apical bristles. Hind tibia with two apical bristles. Hind tarsomere I somewhat shortened and flat. Tibiae and five tarsomeres of legs LI : 2.5 : 1.1 : 0.5 : 0.3 : 0.2 : 0.4; LII : 3.6 : 1.3 : 0.8 : 0.5 : 0.3 : 0.4 ; LIII :3.5 : 0.8 : 1.0 : 0.8 : 0.4 : 0.5. Wing nearly hyaline, tinged light yellow; veins light brown, R_4+5_ and M parallel. CuAx ratio 0.2. Squama pale with long pale hairs. Halter pale.

**Figure 4.**
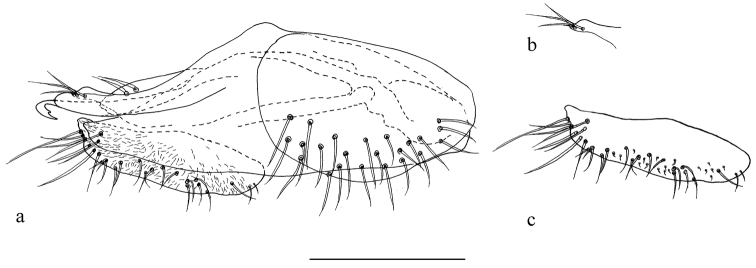
*Protomedetera biseta* sp. n. holotype male, **a** terminalia lateral view **b** apex surstylus **c** cercus. Scale bar: 0.1 mm.

*Abdomen* metallic green with grey pollinosity. Hairs and bristles black. Hypopygium (Fig. 4a): Hairs and bristles pale. Epandrium nearly rectangular, three times as long as wide; epandrial lobe faded with two spinous bristles. Surstylus (Fig. 4b) simple, with three long spine-like bristles. Cercus (Fig. 4c) nearly oval, 3.6 times as long as wide, narrow at tip, with several long apical marginal bristles and some short peg-like bristles. Hypandrium simple, rounded apically in lateral view. Phallus thin, hook-like apically in lateral view, with one preapical denticle.

***Female.*** Unknown.

#### Etymology.

The name *biseta* refers to the two long bristles on the pedicel.

#### Material examined.

Holotype male: PAPUA NEW GUINEA, Madang province, Baiteta (4°59'10.36"S, 145°45'47.05"E), FOG AR 40, 3/VIII/1995, leg. Olivier Missa (in coll. RBINS).

Paratypes: PAPUA NEW GUINEA, Madang province, Baiteta (4°59'10.36"S 145°45'47.05"E), 2 males, FOG AR 1, 27/IV/1995, leg. Olivier Missa; 1 male, FOG AR 15, 15/VI/1995; 1 male, FOG XA 9, 9/IV/1993, leg. Olivier Missa (in coll. RBINS).

### Protomedetera glabra sp. n.

Taxon classificationAnimaliaDipteraDolichopodidae

http://zoobank.org/220EADF8-B451-42D5-9E68-AB0E8D566F87

Figs 5–6 , 7–8

#### Diagnosis.

Pedicel with circlet of short bristles nearly as long as pedicel. Wing veins without any thickness. Cercus rhomboid, with row of long marginal bristles and one long apical bristle, covered by short weak peg-like bristles. Hypandrium simple, black apically.

#### Description.

**Figures 5–6.**
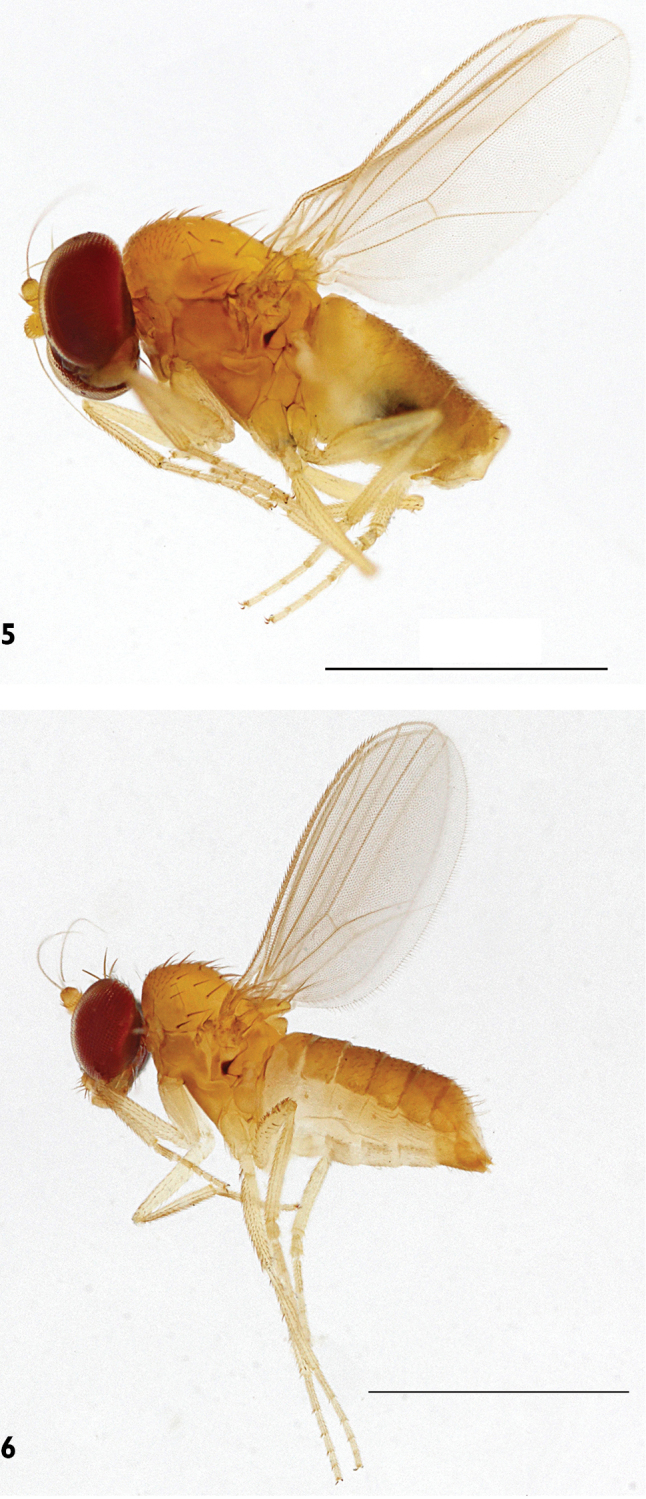
*Protomedetera glabra* sp. n.: **5** Holotype male, habitus, lateral view **6** Female habitus. Scale bar 1: mm (photo credit Ms Chufei Tang).

***Male*** (Fig. 5). Body length 1.3 mm, wing length 1.1 mm, wing width 0.6 mm.

*Head* metallic green, nearly black, with grey pollinosity; eyes separated; face nearly parallel, width of face at middle 0.6 times as wide as first flagellomere. Hairs and bristles on head black, postocular bristles and posteroventral hairs short pale. Two strong oc, two strong vt. Antenna wholly yellow except first flagellomere brownish at apex, scape and pedicel yellow; scape short and small, almost invisible; pedicel cup-like, large, with first flagellomere sunken in it, with circlet of short bristles nearly as long as pedicel; first flagellomere small, rounded, nearly as long as wide, with tiny pubescence; arista black, almost bare, nearly as long as head, with basal segment extremely short, less than 0.1 times length of apical segment. Ocellar tubercle distinct but not strongly raised. Upper postcranium deeply concave. Proboscis yellow, not strongly sclerotized, with light thin lines; palpus black, small, with black preapical bristle.

*Thorax* raised dorsally at front area, dark yellow without metallic gross, brownish at propleuron and mesopleuron, with some grey pollinosity. Mesonotum flat before scutellum. Hairs and bristles on thorax pale; two h, one ph, one su, two npl, two sa, one pt, two weak dc, six biseriate acr at anterior 1/2. Shoulder portion densely covered with short bristles. Scutellum with one pair of strong sc.

*Legs* wholly yellow without distinct bristle. Hind tarsomere I somewhat shortened and flat. Tibiae and five tarsomeres of legs LI : 3.0 : 0.8 : 0.5 : 0.5 : 0.4 : 0.5; LII : 3.5 : 1.3 : 0.8 : 0.6 : 0.4 : 0.5 ; LIII :4.5 : 0.8 : 1.2 : 1.0 : 0.5 : 0.6. Wing nearly hyaline, tinged light yellow; veins light brown, R_4+5_ and M parallel. CuAx ratio 0.2. Squama pale with long pale hairs. Halter pale.

**Figures 7–8.**
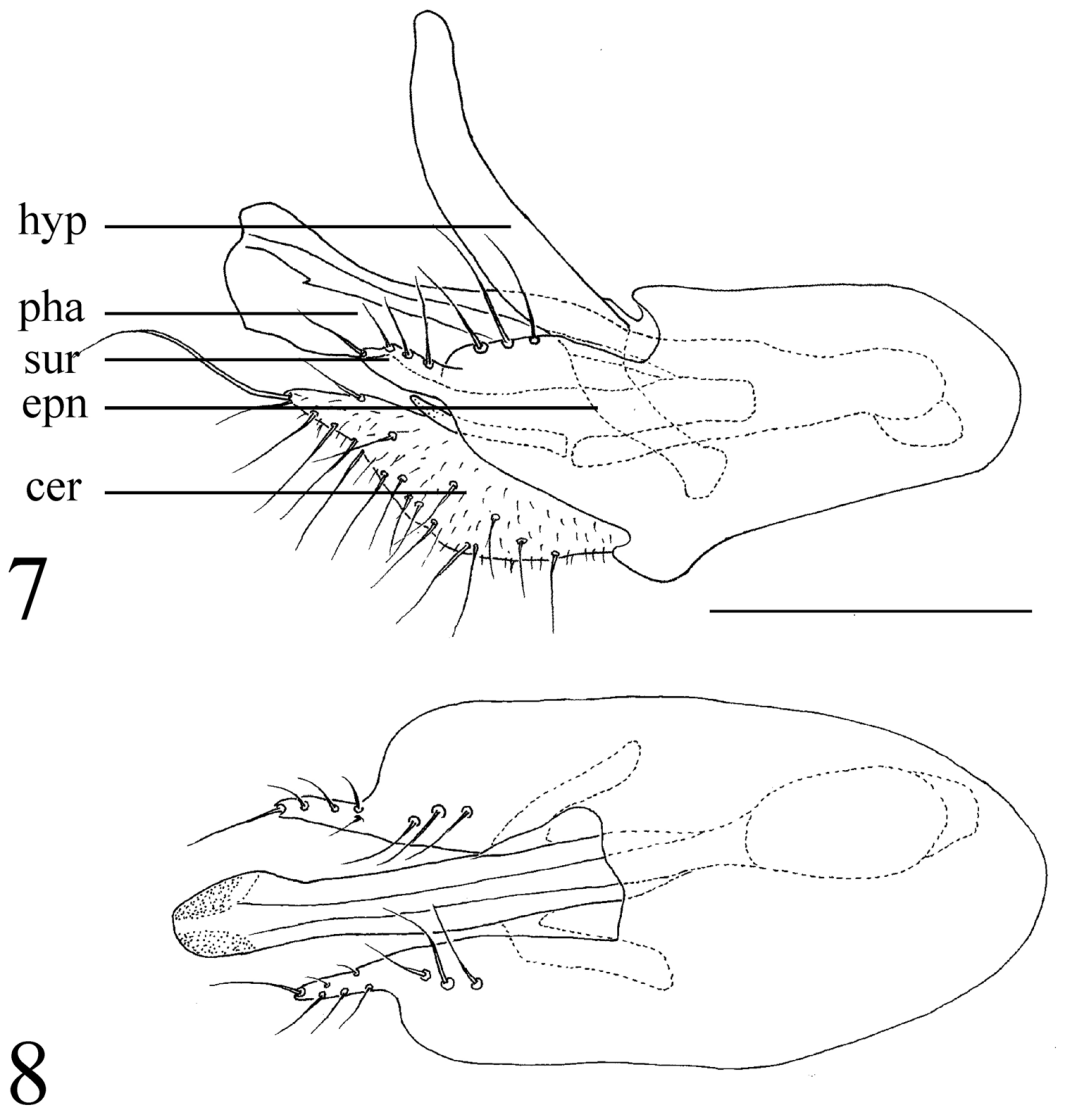
*Protomedetera glabra* sp. n. holotype male: **7** Terminalia lateral view **8** Terminalia dorsal view. Scale bar: 0.1 mm. Abbreviations: epn = epandrium, hyp = hypandrium, pha = phallus, sur = surstylus, cer = cercus.

*Abdomen* metallic green with grey pollinosity. Hairs and bristles black. Hypopygium: simple, pale. Hairs and bristles pale. Epandrium (Figs 7–8) nearly rectangular, about twice as long as wide, with three long bristles; epandrial lobe indistinct but left three long bristles. Surstylus simple, with four bristles. Cercus rhomboid, with row of long marginal bristles and one long apical bristle, covered by short weak peg-like bristles. Hypandrium simple. Phallus inflate and dark apically.

***Female*** (Fig. 6). Almost same to male, but body length 1.5 mm, wing length 1.2mm, wing width 0.7 mm.

#### Etymology.

The name *glabra* means bare, referring to the pedicel which has no long bristles and a simple hypandrium.

#### Material examined.

Holotype male: PAPUA NEW GUINEA, Baiteta (4°59'10.36"S, 145°45'47.05"E), FOG AR 14, 14/VI/1995, leg. Olivier Missa (in coll. RBINS).

Paratypes: PAPUA NEW GUINEA, same provenance as holotype: 1 male, FOG AR 13, 9/VI/1995; 1 male, FOG AR 9-3, 26/V/1995; SINGAPORE: 2 males, Sime forest, 8/IV/2005, leg. P. Grootaert (in coll. LKCNHM); 1 male, Clementi Woods, 23/IV/2005, drains, leg. I. Van de Velde; 1 female, Nee Soon, 27/IV/2005, leg. P. Grootaert; 1 male, Clementi woods, 25/VI/2005, drains, leg. I. Van de Velde (in coll. RBINS).

### Protomedetera singaporensis

Taxon classificationAnimaliaDipteraDolichopodidae

Grootaert & Tang sp. n.

http://zoobank.org/2101D6AC-F4CC-462E-8319-B1E4CA829942

Figs 9 , 10

#### Diagnosis.

Width of face at middle 1.4 times as wide as first flagellomere. Pedicel with only one long black apical bristle 1.5 times as long as total length of pedicel and first flagellomere. Surstylus with four long apical bristles. Cercus with one long apical bristle.

#### Description.

**Figure 9.**
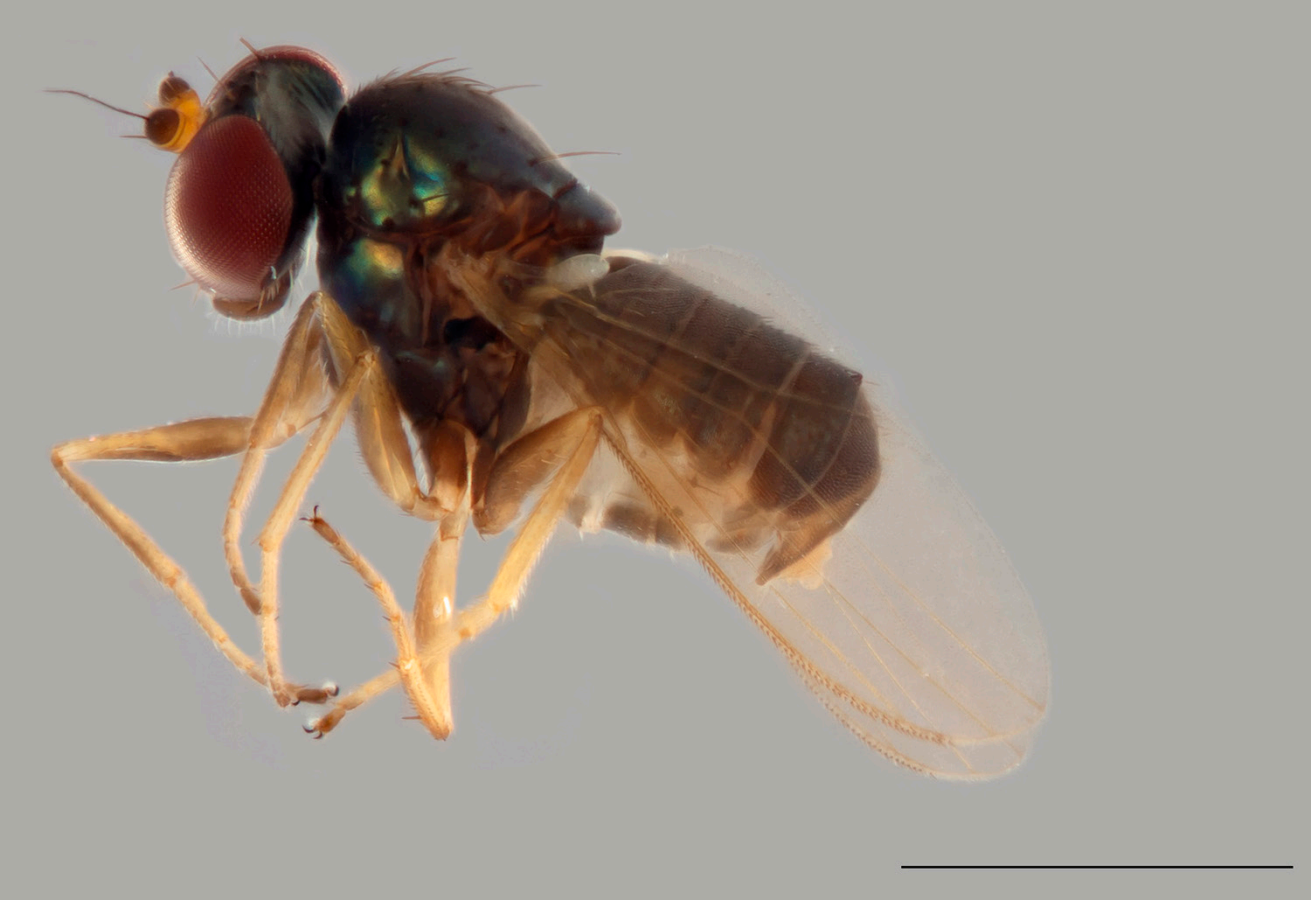
*Protomedetera singaporensis* sp. n. holotype male habitus lateral view. Scale bar: 1 mm. (photo credit Ms Kai Qing Chin).

***Male*** (Fig. 9). Body length 1.8 mm, wing length 1.8 mm, wing width 0.6 mm.

*Head* metallic green, nearly black, with grey pollinosity; eyes separated; face nearly parallel, width of face at middle 1.4 times as wide as first flagellomere. Hairs and bristles on head black except postocular bristles and posteroventral hairs pale. Two strong oc, two strong vt. Antenna dark yellow except first flagellomere black; scape short and small, almost invisible; pedicel cup-like, large, with first flagellomere sunken in it, with circlet of bristles, all yellow and short except one black and long, 1.5 times as long as total length of pedicel and first flagellomere; first flagellomere small, rounded, nearly as long as wide, pubescent; arista black, pubescent, nearly as long as head, with basal segment extremely short, less than 0.1 times length of apical segment. Ocellar tubercle distinct but not strongly raised. Upper postcranium deeply concave. Proboscis brown with brown apical hairs; palpus brown, small, with strong brown apical bristle.

*Thorax* raised dorsally at front area, dark metallic green, with some grey pollinosity. Mesonotum flat before scutellum. Hairs and bristles on thorax pale; two h, one ph, one su, two npl, two sa, one pt, five dc, six biseriate acr at anterior 3/5 portion. Shoulder portion densely covered with short bristles. Scutellum with one pair of strong sc.

*Legs* mainly yellow but coxae, basal 4/5 of femora and tarsomere V brown. Hairs and bristles on legs pale. Bristles on legs thin and hair-like. Fore and mid coxae each with two rows of anterior bristles. Hind femur with one preapical pv. Mid tibia with one ad at basal 1/3 and one short apical bristle. Hind tarsomere I somewhat shortened and flat. Tibiae and five tarsomeres of legs LI : 2.4 : 1.0 : 0.5 : 0.4 : 0.3 : 0.5; LII : 3.5 : 1.2 : 0.8 : 0.5 : 0.3 : 0.4 ; LIII :4.5 : 1.7 : 0.8 : 0.5 : 0.3 : 0.5. Wing nearly hyaline, tinged light yellow; veins light brown, R_4+5_ and M parallel. CuAx ratio 0.125. Squama pale with long pale hairs. Halter pale.

**Figure 10.**
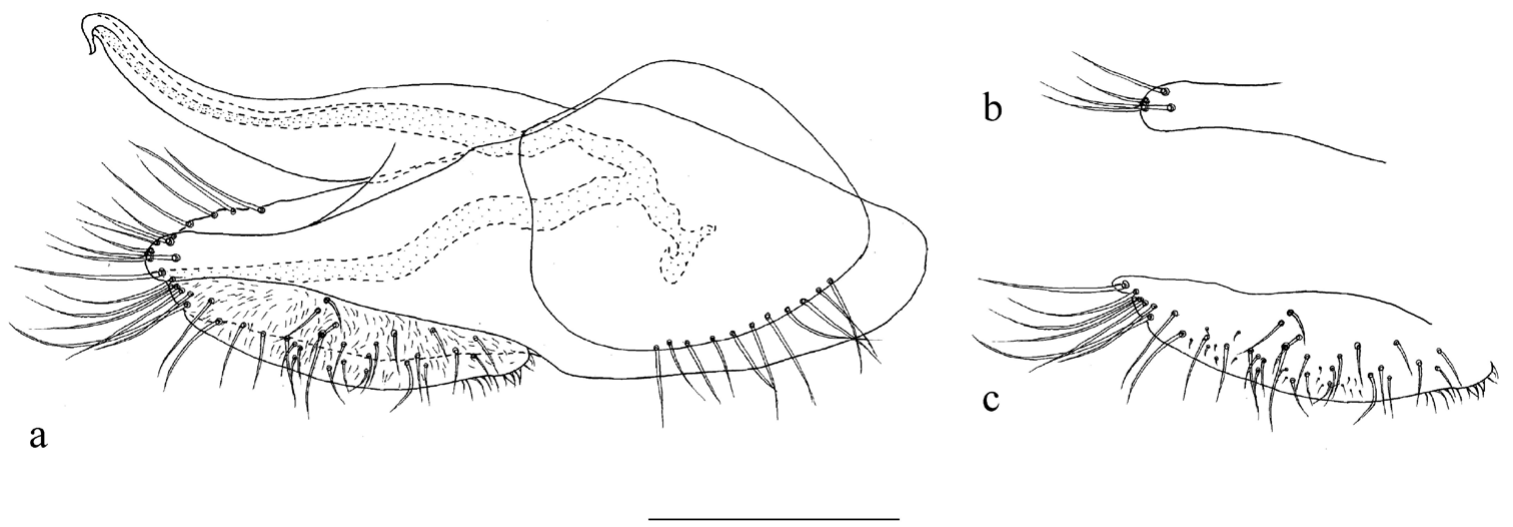
*Protomedetera singaporensis* sp. n. holotype male **a** terminalia lateral view **b** apex surstylus **c** cercus. Scale bar: 0.1 mm.

*Abdomen* metallic green with grey pollinosity except first segment, with one large black spot laterally at segment V. Hairs and bristles black. Hypopygium (Fig. 10a): simple, brown. Hairs and bristles pale. Epandrium nearly rectangular, about 2.5 times as long as wide; epandrial lobe indistinct, only left one row of four long apical bristles. Surstylus (Fig. 10b) simple, finger-like, fused with epandrium, with four long spine-like apical bristles. Cercus (Fig. 10c) nearly oval, 3.2 times as long as wide, slightly narrow at tip, with row of long marginal bristles at apical half, covered by short weak peg-like bristles Hypandrium simple, narrowed towards tip. Phallus simple but hooked apically, mainly hidden in hypandrium.

***Female.*** Unknown.

#### Etymology.

The name *singaporensis* refers to the Republic of Singapore where this species was found.

#### Material examined.

Holotype male: SINGAPORE, Nee Soon (swamp forest), 2 May 2012 in Malaise trap NS2 (leg. J. Puniamoorthy; in coll. LKCNHM with reference R_ckq_DOL_ZRCBDP00005503_Protomedetera_sp_M_NS2).

The NGS barcode is deposited in GenBank accession number: BankIt2080950 Seq1 MG820472.

## Discussion

The concept “microdolichopodid” by Robinson (1975) is used to describe the dolichopodid genera with small body size. Most of the genera included in it belong to Medeterinae. It is useful to compare these genera with each other to find if there is any potential affiliation between them.

Overall, the appearance of *Protomedetera* is similar to *Cryptopygiella* Robinson, they share the small size of the body and the wing, the long bristle on the pedicel, the biseriate acr, the ratio of crossvein dm-cu and distal CuA. However, the male genitalia of the two genera are quite different. In *Cryptopygiella*, the male genitalia are almost completely enclosed in the tip of the abdomen; appendages of male genitalia including the cercus, the surstylus, the epandrial lobes, and the hypandrium are all strongly reduced (Robinson 1975, Runyon et al. 2010). The new genus is also like *Paramedetera* for the small size of body, the similar shape of the epandrium and the cercus of male genitalia as well as the parallel M_1+2_ and R_4+5_, but they are very different from the latter in which the acr are absent. Species of *Paramedetera* also have four dc, but the new species reported here all have five to six dc. Differences also occur in the bristles on the pedicel and the hind tarsomere I. For species of *Protomedetera*, the apical bristles on the pedicel are usually long enough to cover the first flagellomere of the antenna, the hind tarsomere I is short and flat; however, these features are not present in species of *Paramedetera* (Grootaert 2006). Another genus that looks like *Protomedetera* is *Demetera* Grichanov but it can be distinguished from the latter as the hind tarsomere I of *Protomedetera* is shortened and the cercus is usually simple. In *Demetera*, the tarsomere I is normal and the cercus has an elongate ventrolateral arm (Grichanov 2011).

*Protomedetera* is included into Medeterinae for the following features: the flat vertex, the deeply concave upper postcranium, the suboval first flagellomere and the apical arista, the parallel sided face, the pollen on the face obscuring the metallic ground-colour, the strongly flattened and slightly depressed posterior mesonotum, the absent lateral sc, the absent preapical bristles of mid and hind femora, vein M without bosse alaire. Though the facets of eyes do not have any soft hair and the surstylus is not complex, the two features are absent in some other genera of Medeterinae, too. For example, *Medetera* is bare between facets (Bickel 1986a, b, Runyon 2010). Overall, most of the features of *Protomedetera* accord with Medeterinae. Another evidence for our conclusion is the anatomy of the proboscis. Though the structure is not strongly sclerotized, we could see small denticles on the strips of the proboscis which is identical to all the Medeterinae we know. Thus, the new genus is placed as a new member of Medeterinae. The habitat of the genus is similar to all the Medeterinae, too. Species are observed to stay on tree trunks, with the body in an acute angle on the vertical substrate.

The new genus seems to have a wide tropical Oriental and Australasian distribution. It is actually known from Malaysia, Singapore, and Papua New Guinea. Daniel Bickel (pers. comm.) mentioned that it is present in Fiji as well. Since we did not see any differences between specimens of *Protomedetera biconvexa* sp. n. from Papua New Guinea and Malaysia, we consider them as conspecific for the moment. Similar consideration was taken on *P. glabra* sp. n., which distributes in both Papua New Guinea and Singapore. Molecular techniques will be needed in the future to support this conspecifity since such a disjunct distribution is very unlikely.

## Supplementary Material

XML Treatment for Protomedetera

XML Treatment for Protomedetera bico nvexa

XML Treatment for Protomedetera biseta

XML Treatment for Protomedetera glabra

XML Treatment for Protomedetera singaporensis
